# Editorial: The human foot: function in progress

**DOI:** 10.3389/fbioe.2023.1245069

**Published:** 2023-07-17

**Authors:** Karl T. Bates, Madhusudhan Venkadesan, Evie E. Vereecke, James P. Charles, Kristiaan D’Août

**Affiliations:** ^1^ Department of Musculoskeletal and Ageing Science, Institute of Life Course and Medical Sciences, University of Liverpool, The William Henry Duncan Building, Liverpool, United Kingdom; ^2^ Department of Mechanical Engineering and Materials Science, Yale University, New Haven, CT, United States; ^3^ Department of Development and Regeneration, KU Leuven Campus Kulak, Kortrijk, Belgium

**Keywords:** foot and ankle, biomechanics, gait, evolution, bipedalism, modelling

The human foot is arguably our most distinctive morphological and functional structure, and as such is widely considered a defining “hallmark” of our bipedalism. Our closest living relatives, chimpanzees and bonobos, have long toes and a highly mobile mid-foot to provide the necessary flexibility for grasping and climbing in arboreal environments. By contrast, the human foot is unique among great apes in possessing stabilized longitudinal arches in the mid-foot, which impart the necessary stiffness to allow the metatarsals to act as a propulsive lever to enhance the mechanical efficiency of striding bipedalism over relatively uniform terrestrial substrates. In recent years, increased sophistication of biomechanical studies has begun to refine this basic mechanical model of human foot function, noting high levels of anatomical and functional variation within and between human populations and the “tuneable” functionality of the foot in a variety of contexts (e.g., footwear, terrain, behavior). These developments have not only furthered our understanding of the basic mechanical function of the foot, but delivered new insights into selective pressures that have driven our evolutionary history, how the foot is impacted by aging and disease, and how we might better tackle limb dysfunction through clinical treatment.

The goal of the Research Topic “*The human foot: function in progress*” was to further advance our understanding of how anatomy and function interact and contribute to the mechanics and energetics of human locomotion. The authors contributing to this topic present a diverse array of imaging, experimental and computational modelling approaches in pursuit of this goal. Ito et al. uses three-dimensional finite element models to investigate causal relationships present in the disparate anatomy, motion and kinetics of human and chimpanzee feet. Among a number of interesting findings, the models of Ito et al. highlight the contribution of coupling mechanisms between the shank and foot bones to stable bipedalism, and great propensity for the human foot to utilize elastic energy in soft tissue to generate propulsive forces during the stance phase. Three of the contributions in this Research Topic (Matsumoto et al., Sun et al., Welte et al.) focus specifically on the contribution of passive elastic structures (notably the plantar aponeurosis) to foot function, and how their contributions causatively interact with joint kinematics at the ankle and/or within the foot itself. The analysis of Welte et al. similarly emphasises the importance of tuning of elastic structures in the foot to upright bipedal locomotion in humans. These authors use high-speed biplanar x-ray measurements of foot bone motion during walking and running to show that recoil of mid-foot arches lengthens ground contact times and promotes favourable propulsive conditions at the ankle for upright walking on an extended leg (versus more flexed limb postures in non-human apes). They offer interesting discussion of how their findings might stimulate a variety of related research fields, including biological anthropology, footwear and clinical sciences. Sun et al. also use x-ray imaging to quantify foot bone motions, how these vary in different foot strike patterns during running, and subsequently how variations impact upon loading of the plantar aponeurosis. They describe interesting differences in bone motions and plantar aponeurosis mechanics in rearfoot strike versus forefoot strike running conditions, and suggest (among other interpretations) that forefoot striking may increase the storage and release of elastic energy in the midfoot and its associated soft tissue structures, like the plantar aponeurosis. Matsumoto et al. use a similar digital modelling framework to Ito et al. and demonstrate the capacity of “virtual” or *in silico* approaches to provide functional insights in circumstances where invasive experimentation is not feasible. Through model parameterisation they explore the contribution of the plantar aponeurosis to mechanical work at different joints within the foot, which again emphasises the importance of quantifying the impact of elastic structures on foot function in both basic science and clinical contexts. The importance of a deeper scientific understanding of basic foot function is emphasised by Michels et al. in their review article focused on subtalar joint instability. They highlight that poor understanding of the relative contributions of intrinsic ligaments to subtalar joint stability and limited diagnostic procedures represent long-standing obstacles in clinical conceptions and treatment of subtalar instability. Michels et al. subsequently review how recent biomechanical studies of the subtalar joint and its associated soft tissues have improved the understanding of pathophysiology on diagnosis and treatment. Finally, Papachatzis et al. present a novel investigation of the relationship between the dissipative energetic properties of the heel and its thermodynamic responses during walking. They test hypotheses related to the interaction between mechanical and thermodynamic functioning of the heel during foot contact by collecting motion and temperature on humans walking with different magnitudes of added weight. They conclude that a complex array of heat dissipation mechanisms must contribute to the heel’s thermodynamics responses during walking, and that additional factors (beside energy dissipation) should be explored in future studies. Together, these papers show that our insight in the functional anatomy of the foot continuously advances thanks to the use of innovative methods, the combination of techniques and expertise from different disciplines, and effective collaboration between researchers.

